# *In Vivo* and *In Vitro* Characterization of a *Plasmodium* Liver Stage-Specific Promoter

**DOI:** 10.1371/journal.pone.0123473

**Published:** 2015-04-15

**Authors:** Mariana De Niz, Susanne Helm, Sebastian Horstmann, Takeshi Annoura, Hernando A. del Portillo, Shahid M. Khan, Volker T. Heussler

**Affiliations:** 1 Institute of Cell Biology, University of Bern, Bern, Switzerland; 2 Molecular Parasitology, Bernhard Nocht Institute for Tropical Medicine, Hamburg, Germany; 3 Center of Infectious Diseases, Leiden University Medical Center, Leiden, The Netherlands; 4 Department of Parasitology, National Institute of Infectious Diseases (NIID), Tokyo, Japan; 5 Barcelona Centre for International Health Research (CRESIB), Barcelona, Spain; 6 Institució Catalana de Recerca i Estudis Avançats, Barcelona, Spain; Université Pierre et Marie Curie, FRANCE

## Abstract

Little is known about stage-specific gene regulation in *Plasmodium* parasites, in particular the liver stage of development. We have previously described in the *Plasmodium berghei* rodent model, a liver stage-specific (*lisp*2) gene promoter region, *in vitro*. Using a dual luminescence system, we now confirm the stage specificity of this promoter region also *in vivo*. Furthermore, by substitution and deletion analyses we have extended our *in vitro* characterization of important elements within the promoter region. Importantly, the dual luminescence system allows analyzing promoter constructs avoiding mouse-consuming cloning procedures of transgenic parasites. This makes extensive mutation and deletion studies a reasonable approach also in the malaria mouse model. Stage-specific expression constructs and parasite lines are extremely valuable tools for research on *Plasmodium* liver stage biology. Such reporter lines offer a promising opportunity for assessment of liver stage drugs, characterization of genetically attenuated parasites and liver stage-specific vaccines both *in vivo* and *in vitro*, and may be key for the generation of inducible systems.

## Introduction

Throughout its life cycle, the *Plasmodium* parasite requires adaptation during the various stages in mosquito and human hosts. The parasite’s regulation of gene expression during all life stages remains incompletely understood, with few transcription factors and stage-specific promoters characterized [1–5]. The liver stage is particularly neglected in this respect despite identification of various liver stage-specifically expressed genes [6–10]. Sporozoites in the liver stage, and merozoites in the blood stage must exploit extremely different host cell types for successful growth and multiplication. Several studies have suggested the existence of stage-specific expressed genes, regulating processes potentially unique to the liver. The specific function of most of these liver stage-specific genes, however, remains unknown. More importantly, there is still a major lack of understanding on how gene expression is regulated in pre-erythrocytic stages. In this context, liver stage-specific genes may be key for identifying and/or validating targets for attenuated vaccines, and liver-stage anti-malarial drugs. Therefore, the development of tools that enable studying and manipulating liver stage-specific gene regulation continues to hold promising potential in the field.

In search of a better understanding of gene regulation and protein expression at the liver stage, our lab previously identified and characterized a 989bp liver stage-specific promoter region PB103464.00.0 (PBANKA_100300) [11]. More recently this gene and its gene product have been described by others,. They were also given the name LISP2 for liver-specific protein 2, and shown to belong to the *Plasmodium* 6-Cys family [10, 12]. In these studies, *lisp2* was confirmed to be liver stage-specific, and proven to be key for late liver stage development [12], confirming our previous findings [11]. Henceforth, in our present work, we refer to the PB103464.00.0 promoter we previously characterized, as the *lisp2* promoter.

To enable quantification of promoter activity, we made use of a dual luciferase reporter system with *Renilla* luciferase expressed under the control of the constitutive *ef1α* promoter, and firefly luciferase expressed under the control of the 989 bp liver stage-specific *lisp2* promoter region. A major advantage of bioluminescence reporters is the possibility to measure real time kinetics of cell movement, gene expression patterns, transcriptional promoter activities, protein-protein interactions, protein conformational changes, and cell signaling, among other biomolecular activities in living animals [13]. At the same time, bioluminescence imaging is also a valuable tool to study host-pathogen interactions [14]. In *Plasmodium* research, the introduction of bioluminescence into rodent models has enabled characterization of *P*. *berghei* and *P*. *yoelii* development in blood [15–18], and pre-erythrocytic stages [19–24], including evaluation of the effects of irradiation and anti-malarial prophylaxis on liver-stage parasite development.

Bioluminescent reporters are of a non-invasive nature and offer the possibility of spatio-temporal analyses within the same organism. Previously, however, a drawback associated with bioluminescence as opposed to fluorescence, was the limited versatility of reporters in terms of light-emitting spectra. Recent advances now enable multiplexing approaches to measure multiple parameters in the same sample. Key developments include the possibility of combining luciferases with non-overlapping spectra and/or substrate requirements such as firefly and *Renilla* (with peak luminescence at 562nm and 480nm respectively, and D-luciferin and coelenerazine substrate requirements respectively). Mutation studies have led to variants with greater spectral diversity and stability for each reporter. These include, for instance, red-shifted variants of *Renilla* luciferase [25], and green and red-shifted variants of Firefly luciferase [26]. The dual bioluminescence reporter system in our study allowed us to measure two parameters simultaneously in the same sample, namely infection rate via the activity of a constitutive promoter, and liver stage-specific promoter activity. Since our transfection plasmid contains both, Renilla and Firefly luciferase-coding sequences, it is not necessary to clone the newly generated parasite population. Cloning by limiting dilution needs many mice and since our approach avoids this, it was reasonable to generate numerous deletions and mutations of the *lisp2* promoter region as performed in this study. In general, multiplex approaches have previously proven to be valuable tools to maximize readouts while minimizing time, costs, and animal use in *in vivo* studies [27–32].

While our previous work led to successful characterization of the promoter region *in vitro*, we show here a characterization of its activity *in vivo*, and evaluate its potential for studying liver-stage specific targets in live rodent models. In addition, we further characterized the promoter region, particularly in the elements that define stage-specificity. Although this particular promoter region does not necessarily encompass the entire gene promoter activity, we have explored this liver stage-specific promoter region and characterized it in detail. We present it here as a promising tool for liver-stage research enabling stage-specific protein expression, modification, and attenuation.

## Materials and Methods

### Experimental animals

Female BALB/c mice, 4–6 weeks old, weighing 20 to 30g at the time of infection were used. Animals were kept at an S2 facility. All studies in which animals were involved, were performed in accordance with the regulations created and approved by the Animal Research Ethics Committee of the Canton Bern, Switzerland (Permit Number: 81/11 and 105/10), and the University of Bern Animal Care and Use Committee, Switzerland. All measurements were performed under isofluorane anesthesia, and all efforts were made to minimize suffering.

### Generation of transgenic *P*. *berghei* parasites

Transgenic *P*. *berghei* parasites PbFL_lisp2_RL_ef1α_ , PbFL_ef1α_RL_ef1α_, PbFL_lisp2(-775)_RL_ef1α_, PbFL_lisp2(-318)_RL_ef1α_, and PbFL_lisp2(_*_825)_RL_ef1α_ have been described previously [11]. All new parasite strains were generated using similar plasmids and methods. pFL_lisp2-AMA1_RL_ef1α_ and pFL_lisp2-αTub1_RL_ef1α_ were generated by substitution of the 5’UTR of the *lisp2* (-318/+1) promoter region with the 5’UTR of the stage-specific AMA1 gene (PBANKA_091500), or the 5’UTR of constitutively-expressed α-tubulin (PBANKA_041770) respectively, fused to the remainder of the promoter. The 5’UTR of AMA1 and α-tubulin was determined by analyzing the corresponding EST found in the PlasmoDB database and by comparison with the *P*. *falciparum* homologs. The 5’UTR of the α-Tubulin gene was amplified with primers F1: 5’- CGCCTAGGATACATTATTTAAATAAATGAAATTGAGAGTATTAT-3’ and R1: 5’- CGCCTAGGTTTACTTGTATATTATAAAATAAACAATTGTTTTTA-3’, while the 5’UTR of the AMA1 gene was amplified using primers F2: 5’-CGCCTAGGCGTACATCTACGCATTGTTATTTAGC-3’ and R2: 5’- CGCCTAGGTTTTTATATCGTTTTATTTTATTAATATTTTTAATTTAC-3’. These were then cloned into the dual luciferase pFL-989/-318RLef1α plasmid, via enzymes AvrII and BamHI, and transfected into *P*. *berghei* to generate the parasite lines PbFL_lisp2-AMA1_RL_ef1α_ and PbFL_lisp2-αTub1_RL_ef1α_.

### RNA isolation and qRT-PCR

Total RNA was isolated from infected hepatocyte cultures using the NucleoSpin RNA kit (Macherey & Nagel), according to manufacturer’s instructions. To examine gene expression on the RNA level, cDNA was synthesized. The starting material was ~1μg RNA dissolved in RNAse-free water; 3μl of 50μM random primer solution, and 1μl 10μM dNTPs to a final volume of 12μl complemented with distilled water. The mixture was heated for 5min at 65°C and then cooled on ice for 2 minutes. To the mixture, 4μl of 5x First Strand Buffer for reverse transcriptase, 10mM DTT, 1.5μl of distilled water and 100 U Reverse Transcriptase SuperScript II were added. cDNA synthesis was carried out for 50min at 42°C. To inactivate the reverse transcriptase, the mixture was heated to 72°C for 15min. Samples were afterwards stored at -20°C. To detect genomic DNA contamination, samples without reverse transcriptase were run in parallel in all experiments.

To quantify the amount of mRNA of LISP2 during the liver stage, quantitative real-time PCR was performed using the SYBR ROX Master Mix Kit (5 PRIME). For LISP2 profiling, forward primer 5’AACAGCAATATATCGTCACCAAG 3’ and reverse primer 5’ TGCAAAGGTAATTATTAGAAATCGT 3’ were used. Normalization was performed using the *P*. *berghei* 18S ribosomal RNA using forward primer 5’ GGATGTATTCGCTTTATTTAATGCTT 3’ and reverse primer 5’ CACGCGTGCAGCCTAGTAT 3’. In a reaction volume of 20μl, 10pmol primers and 25ng of cDNA were added. All assays were run in duplicate. PCR cycles for the Rotor-Gene RG-3000 (Corbett Research) were 1x 30s 95°C; 1x 2min 95°C; 15s 95°C, 20s 50°C, and 20s 68°C for 35x; and 1x 2min 95°C. Changes at the level of RNA expression were calculated using the Rotor-Gene software 6.0.

### Parasite growth and sporozoite isolation

4–5 week-old Balb/c mice were injected with blood stage transgenic parasites. At day 3–4 post-infection upon first observation of gametocyte exflagellation in the blood, the infected mice were used to feed *Anopheles stephensi* female mosquitoes. 10–11 days following the blood-meal, 15–20 mosquitoes were dissected and the midguts isolated to evaluate oocyst formation and luciferase expression. At day 16–26 post-blood meal, salivary gland sporozoites were isolated in order to perform *in vitro* and *in vivo* infections as described below, as well as luciferase expression assays.

### Hepatocyte culture and *in vitro* infection assays

Human hepatoma HepG2, Hepa1–6 and Huh7 cells (European Collection of Cell Culture) were maintained in complete MEM (cMEM) containing Earle’s Salts Medium, complete DMEM medium, or RPMI 1640 respectively, supplemented with 10% heat-inactivated foetal calf serum (FCS), 1% L-Glutamine, 1% penicillin/streptomycin. Cells were kept at 37°C in a 5% CO_2_ cell incubator and were split every 4 days by treatment with acutase. 5x10^4^ cells were seeded into 24-well plates. Sporozoites were prepared from dissected salivary glands, incubated in complete MEM, DMEM or RPMI 1640 containing Amphotericin B (2.5μg/ml), and added to 24 well-plates containing monolayers of HepG2, Hepa1–6 or Huh7 cells respectively. Following washing, the cells were incubated with AT-medium at 37°C and 5% CO_2_ for the indicated times.

### Mouse infection assays

BALB/c mice were infected with PbFL_lisp2_RL_ef1α_ or PbFL_ef1α_ parasites either by intravenous injection of salivary gland sporozoites, or by infectious mosquito bites. In the case of intravenous injections, salivary gland sporozoites were counted in a Neubauer chamber, and either 10^4^, 10^5^ or 10^6^ were re-suspended in 200μl of 1x PBS, and injected into each mouse via tail vein injection. To perform mosquito bite infections, mice were anaesthetized using Ketamine/Xylazine, and placed on a feeding cage containing 30 mosquitoes, for no more than 10 minutes.

### Assessment of luciferase activity

#### *In vitro* dual-luciferase assays

To visualize liver stage luciferase expression in the transgenic *P*. *berghei* lines, infected Huh7 cells were processed following manufacturer’s instructions at 7, 24, 30, 40, 48, 54, and 65 hours post infection. Briefly, the culture medium was removed, wells were washed once with 1x PBS, and lysed for 15min at 30°C with 100μl of 1x Passive Lysis Buffer (PLB) obtained from the Dual-Luciferase Reporter (DLRTM) Assay System (Promega). After centrifuging the samples for 30s at 12,000g, 20μl of the Huh7 cell lysate was transferred to a black 96-well plate (Greiner bio-one) for immediate measurement, or stored at -20°C until use. 100μl of luciferase assay reagent II (LAR-II) was added for measurement of firefly luciferase activity using the In Vivo Imaging System IVIS Lumina II, (Caliper Life Sciences). Following a 10s exposure, 100μl of Stop&Glo were added to the wells, to quantify renilla luciferase activity. *In vitro* measurements were done using a height of 7.5cm (FOV-B), a medium binning factor [8], and 10–20s exposure times. The quantitation of luciferase activity was performed using the Living Image 4.1 software (Caliper Life sciences) to obtain RLU values. The mean RLU value of control samples was subtracted from the mean RLU value of every infected time point. The ratio of the firefly and *Renilla* luciferase RLUs was indicative of the activity of the promoter of interest.

#### Real time *in vivo* bioluminescence imaging of liver and blood stage development

Luciferase activity *in vivo* was determined via full body imaging of mice using the IVIS Lumina II imager. Infected Balb/c mice were anaesthetized using the isofluorane anaesthesia system (XGI-8, Xenogen, Caliper Life Sciences). Measurements were performed in mice infected with PbFL_lisp2_RL_ef1α_, PbFL_ef1α_ and PbRL_ef1α_, at 24, 36, 40, 42, 44, 48, 56, 65, 72, and 90h post-infection. For final firefly quantitation, anaesthetized mice were injected with 150μl of RediJect D-Luciferin (30mg/ml; Perkin Elmer) intraperitoneally. Mice were kept anaesthetized during the measurements, performed within 6 to 12 minutes after injection of the substrate. Following quenching of the firefly signal, renilla quantitation was performed. RediJect Coelenterazine-h bioluminescent substrate (Perkin Elmer) was injected intravenously into the tail vein (100μl/mouse), and measurements were performed immediately given the high quenching rate of the luminescent signal. Bioluminescence imaging was acquired with a 10cm FOV (C), medium binning factor, and an exposure time of 3 to 5 minutes. Quantitative analysis of bioluminescence was performed by measuring the luminescence signal intensity using the Living Image 4.4 software, expressing values in ‘photons’ for both luciferases to establish baseline relative luminescence ratios (FL/RL). Luciferase activity in individual organs (heart, liver, spleen, lungs, and adipose tissue) was visualized in organs dissected 48 and 68h after sporozoite injection or mosquito bite to confirm stage-specific activity.

## Results and Discussion

### *Lisp2* promoter region activity during *in vitro* liver-stage development

Quantitative analysis of liver stage development and promoter activity *in vivo* and *in vitro* is difficult due to the low numbers of infected host hepatocytes. Luciferase assays have proven to be an extremely useful tool, advantageous in terms of time requirements per experiment, and quantitation accuracy [20, 24]. The Dual-Luciferase Assay (DLA) system enabled us to perform simultaneous monitoring of expression of the two luciferase proteins renilla and firefly, due to their divergent bioluminescent emission wavelengths. A major advantage of multi-reporter bioluminescence in general, and the DLA system in particular, is that it enables quantification and comparison of levels of expression of each reporter protein independently in the same sample, thus reducing experimental error. Previous studies using the DLA system have emphasized, among its advantages, high efficiency, accuracy, reproducibility and high reflection of promoter activity [33].

**Fig 1:**
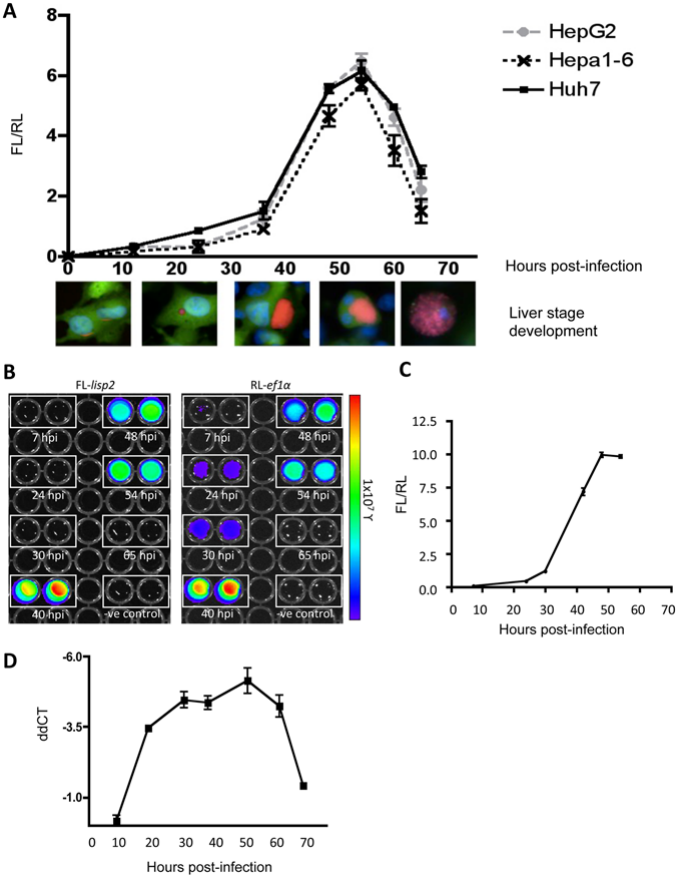
Assessment of the *lisp2* promoter activity *in vitro*. (A) HepG2, Hepa1-6 and Huh7 cells were infected with transgenic PbFL_lisp2_RL_ef1α_ sporozoites and lysed at various time points post-infection (12h, 24h, 36h, 48h, 54h, 65h). *lisp2* activity respective to ef1α (FL/RL ratio) at all time points is depicted. Images from IFAs of the corresponding developmental stages for each time point show *P*.*berghei* mCherry-expressing parasites (red), GFP-transfected HepG2cells (green) and DAPI labeling the host and parasite nuclei (blue). (B) Dual-luminescence assays (DLA) and visualization of infected Huh7 cells using the IVIS Lumina II system. Results for indicated time points are shown for *lisp2* (firefly) or ef1α (renilla) promoter activities. Negative controls correspond to uninfected Huh7 cell lysates. All experiments were carried out in triplicate; error bars correspond to standard deviations. (C) Graphical illustration of the experiment in (B). The FL/RL ratio was calculated and presented over hour post-infection. (D) The transcription profile of LISP2 during the liver stage was determined by qRT-PCR. Total RNA from infected HepG2 cells was isolated at the indicated time points, and then a quantitative real-time PCR analysis was performed. The transcription profile was calculated based on the *P*. *berghei* 18SrRNA control expression, and is indicated in ΔΔCT.

In the DLA system we employed to analyze *lisp2* promoter activity, expression of firefly luciferase was under the control of the liver stage-specific promoter, while renilla luciferase was under the control of the constitutive *ef1α* promoter. Luminescence of firefly and renilla luciferases in cell lysates was determined by two methods, a) micro-plate reader (Fig 1A), and b) IVIS Lumina II system (Fig 1B and 1C). The ratios of firefly to renilla luminescence (FL/RL) of lysates corresponding to Huh7, HepG2 and Hepa1-6 cells infected with *PbFL*_*lisp2*_ *RL*_*ef1α*_ parasites were initially determined by micro-plate reader. A similar FL/RL ratio through the time course of infection was detected in all cell types, as measured from 12 to 65 hours post-infection (Fig 1A). The FL/RL curve revealed no or only very little firefly luminescence by 24hpi. The development of liver trophozoite into schizont stages occurs between 24 and 36hpi; a strong increase in firefly luminescence values was observed from 36hpi onwards, with a maximum intensity detected at 54hpi after which firefly luminescence fell again. At 65hpi, corresponding to merozoite generation and merosome formation preceding blood stage infection, detection of firefly was low. Together it appears that expression *of lisp2* is restricted to the highly proliferating schizont stage. Although FL/RL values remained conserved across the various cell types (Fig 1A), suggesting little host-cell influence in promoter activity, individual luminescence values and luminescence kinetics varied significantly between cell lines (S1 Fig) indicating that infection success and/or parasite maturation in the different cell lines varied considerably.

Luminescence kinetics of infected Huh7 cell lysates measured by the IVIS Lumina II system at various time points (Fig 1B and 1C) largely confirmed the results obtained with the microplate reader. Parallel measurements for firefly luminescence under the control of the *lisp2* promoter region (Fig 1B, left image), and renilla luminescence under the control of the constitutive ef1α promoter (Fig 1B right image) were performed. Firefly luminescence was detected only in the late liver stages (40, 48, and 54 hpi), while renilla luminescence was detected as early as 7h post-infection, and the calculated FL/RL ratio (Fig 1C) was very consistent with the results identified by microplate reader (Fig 1A). Interestingly, at 65h post infection, corresponding to merosome formation, neither firefly nor renilla luminescence were detected. Obviously, in terms of sensitivity the use of a microplate reader has a clear advantage.

To show whether luciferase activity reflects LISP2 activity at the mRNA level, the transcriptional profile of LISP2 was studied via quantitative real-time PCR analysis using the constitutively transcribed *P*. *berghei* 18SrRNA as control. As expected, we observe that the LISP2 mRNA profile reaches a maximum during schizogony, and decreases during merosome formation (Fig 1D).

### Analysis of *lisp2* promoter activity *in vivo*

**Fig 2:**
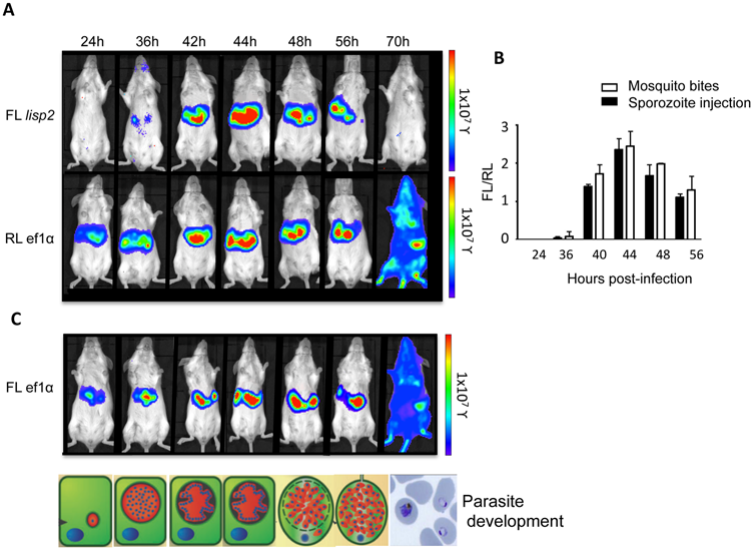
Assessment of the *lisp2* promoter activity by *in vivo* bioluminescence. Balb/c mice infected by intravenous (i.v.) injection with PbFL_lisp2_RL_ef1α_ sporozoites. (A) *lisp2* and ef1α promoter activities were measured by whole body luminescence at the indicated time-points (hours post-infection; hpi). Note that representative pictures of various time points after infection of a single mouse per experiment are shown in A and C. B) ROI measurements for the abdominal area of all mice (n = 9) were recorded as photons per second (photons/s/cm^2^) for renilla and firefly. Ratios of PbFL_ef1α_ to PbRL_ef1α_ were set as normalization value 1 at all time points. Relative luminescence ratios (FL/RL) for all time points are shown. Experiments were repeated 4 times (error bars show SD). (C) Control Balb/c mouse infected by intravenous injection with FL_ef1α_ sporozoites. Bioluminescent measurement of a single mouse at various time points depicted. For a better orientation, a schematic representation of the developmental stage at each time point is shown.

Having confirmed the liver stage-specific activity of the *lisp2* promoter *in vitro*, we next intended to test it *in vivo*. Balb/c mice were infected either by intravenous (i.v.) injection of sporozoites, or mosquito bites with the double luminescent *P*. *berghei* strain PbFL_lisp2_RL_ef1α_.[11]. Luciferase activity in the infected mice was visualized using the IVIS system from 24 to 70 hpi. It is important to note that in Fig 2A, representative images of the separate firefly and renilla luciferase measurements of the same mouse are shown. Average FL/RL luminescence values for both intravenous and mosquito bite injections of nine mice are summarized in Fig 2B. A major strength of the dual luciferase approach is that each image is internally controlled. Firefly luciferase activity (Fig 2A, upper panel) was consistently detected from 42hpi onwards, reaching a maximum at 44hpi, followed by a gradual decrease until 56hpi, and no detectable signal at 70hpi when the liver stage is completed and the blood stage infection has already started (as visualized by constitutive renilla luciferase activity that allows detection of parasites at any given stage in the same mouse) (Fig 2A, lower panel). The peak of firefly luciferase is about 12 hours earlier *in vivo* than it is *in vitro* (Fig 1), which reflects the well known fact that parasite development *in vitro* is delayed compared to the development *in vivo*. Considering this, the calculated ratio of FL/RL *in vivo* (Fig 2B) confirms nicely our *in vitro* observations with a relatively late start of firefly luciferase expression, a fast peak and a rapid drop to undetectable levels, and complete inactivity during the blood stage. Similar FL/RL ratios were observed when mice were infected with PbFL_lisp2_RL_ef1α_ transgenic parasites by mosquito bites instead of intravenous sporozoite injections (Fig 2B). In contrast to the transient and strictly stage-specific firefly activity, renilla luminescence showed a linear increase from 24 to 56h post-infection, followed by a decrease at 65h (data not shown). This was expected as at this time point, liver stage is finished and merozoites first need to establish in red blood cells before the *ef1α* promoter becomes active again and parasitemia reaches a minimum detectable threshold level. At 70h, renilla luminescence was detected throughout the body of the mouse, with strongest signals arising from regions corresponding to the lungs, adipose tissue and spleen, potentially due to sequestration in these organs. The observed renilla activity during blood stage development coincides with an increase in parasite burden (analyzed by stained blood smears) if followed for extended periods (S2 Fig).

Finally, in order to validate that the increased ratio of firefly over renilla luciferase was independent of the luminescent nature of firefly luciferase itself, control animals were infected with a PbFL_ef1α_ single luminescent parasite strain. Luminescence levels during liver-stage development of these parasites are shown in Fig 2C. We found an increase in firefly luciferase activity similar to the renilla luciferase activity detected in PbFL_lisp2_RL_ef1α_ -infected mice (Fig 2A, lower panel) supporting the suitability of the dual-luciferase assay for *in vivo* experiments without an adverse effect of substrate used for this study.

The need for renilla luciferase substrate analog testing and optimal enzyme-substrate pairing for dual luciferase studies *in vivo* has been consistently emphasized in studies making use of the dual (or triple) bioluminescence system. Bioluminescent reporter proteins, like firefly, *Renilla*, or Gaussia luciferases display differences in terms of reporter sensitivity, signal-to-noise ratio, quantitative correlation between signal strength and target numbers, anatomic resolution, and kinetics [34, 35], as well as dependence on the properties of biological tissues being imaged, and substrate delivery methods. Most studies using single bioluminescence reporters emphasize the need to optimize substrate choice and delivery methods in light of the proposed objectives of the study [17, 34–41]. In the case of multi-reporter and multi-component bioluminescent imaging used for comparative quantitation or normalization, optimization of the above features is key. In our study, we have optimized both the administration method and substrate choice. Results on optimization of the DL system for use during *Plasmodium* infection of mice are included in S1 Table. Various substrates for measuring firefly luciferase activity were used, and little variation was found in terms of signal stability, signal duration, and low signal-to-noise ratio. Similarly, little variation was found regardless of the method of administration. Conversely, *Renilla* has been reported in most *in vivo* studies, to present considerable hindrances. Specifically, studies using dual bioluminescence have reported the primary challenge to be the relatively short duration of the renilla luciferase signal [42]. The extremely high noise in the intestinal tract resulting from i.p. or failed intravenous injections and the short duration of the renilla signal requiring immediate imaging, were challenges we faced before achieving direct comparison of the promoter activities. In terms of substrate pairing, the 3 substrates evaluated in our study, namely the Promega second generation substrates EnduRen and Viviren, and the RediJect coelenterazine-h, have all been optimized to counter the above challenges in terms of tissue penetration, noise signals, and duration; for our study, we found RediJect coelenterazine-h to provide the greater balance in terms of efficiency, accuracy and cost, for measuring promoter activity. We thus emphasize the need for optimization in future studies involving the use of a dual or multiplex luciferase assays. Various anesthetics were tested to observe their effects on firefly and renilla luminescence values and FL/RL ratios, given previous observations of detrimental effects of different anaesthetics on substrates including luciferin, but not coelenterazine [43–46].We hypothesize that the lower FL/RL ratios detected *in vivo* compared to *in vitro* may in fact be, to some extent, the effect of anesthetics on inhibition of firefly luciferase. We envisage that ongoing improvements in bioluminescence techniques will improve *Plasmodium* research during the liver stage. These include the generation of red-shifted renilla luciferase which overcome current issues with tissue penetration, as well as the development of electron multiplying and intensified CCD cameras, that enable acquisition times of millisecond durations eliminating the need to use anesthetics which are also hepato-toxic in repeated doses [35]. Despite the above limitations, the dual luciferase system has enabled us to prove again the stage-specificity of the promoter, and the possibility for its use in further *in vivo* studies characterizing *Plasmodium* liver stage vaccine targets and candidate proteins, as well as further examination of biological events.

### Identification of firefly and renilla signals in organ extracts

**Fig 3:**
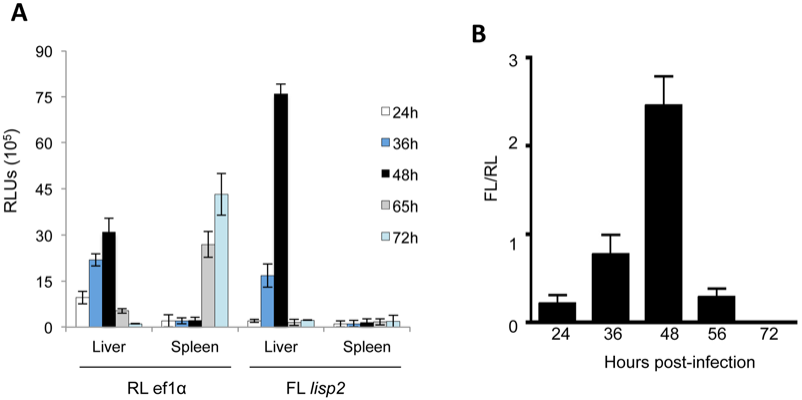
Luminescence in liver and spleen extracts from PbFL_lisp2_RL_ef1α_ infected mice. The liver and spleen from infected and control Balb/c mice were extracted at various time points, and splenocyte and hepatocyte lysates generated. (A) Cells were probed with renilla or firefly substrates and luminescence values obtained by microplate reader. Luminescence values of spleens and livers of infected mice are shown and were normalized with values of uninfected mice. (B) Luminescence ratios (FL/RL) of liver extracts at various time points post-infection as a measure of *lisp2* to ef1α activity ratios.

A known limitation to bioluminescence imaging studies *in vivo*, is the reduced detection sensitivity of signals within deep tissues [47, 48]. To confirm both the liver-stage specific activity of the *lisp2* promoter region, and its increased activity during mid- and late- liver stages found by *in vitro* and *in vivo* analyses, we performed cell extracts of individual organs for determination of luciferase activity in mice infected with PbFL_lisp2_RL_ef1α_ parasites. Following imaging with the IVIS Lumina system of the live mouse, we proceeded to euthanize 3 infected mice at 24, 36, 48, 65 and 72h post-infection, to obtain extracts from the liver and the spleen (Fig 3A and 3B). As control for unspecific luciferase activity, we euthanized naïve mice at each time point and used them to normalize the values obtained from infected mice. Fig 3A confirms that firefly luciferase is strictly liver stage-specific as no signal was obtained in the spleen. The peak of firefly luciferase activity was at 48 hpi similar to what has been determined in the infected living animal. *Renilla* luciferase activity was also detected in both organs with the expected kinetics: until 48 hpi a linear increase in enzyme activity was detected followed by a sharp drop at 65 hpi and at the same time a strong increase in renilla activity in spleen extracts confirming that the switch from liver to blood stage has occurred. Moreover, the switch from liver to blood stages of infection can be clearly observed from the lower luminescence values of renilla (RL_ef1α_) at 65 and 72hpi in the liver, but increasing renilla luminescence in the spleen at the same time points.

### Characterization of the *lisp2* promoter region by deletion analyses

**Fig 4:**
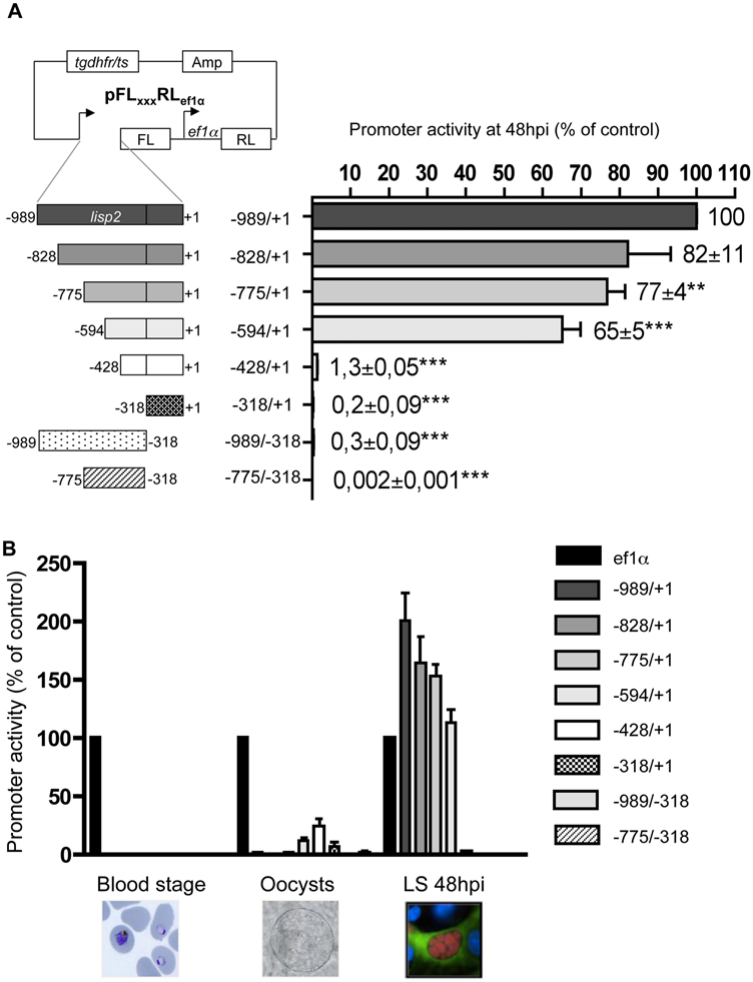
Deletion analysis of the *lisp2* promoter activity at different life cycle stages. (A) Left panel: Schematic representation of promoter regions, each of which was cloned in front of the firefly luciferase gene. Renilla luciferase was constitutively expressed under the ef1α promoter. Right panel: *In vitro* measurements of promoter activity. HepG2 cells were infected with transgenic *P*. *berghei* sporozoites and analyzed at 48h post-infection. The FL/RL ratio of PbFL_lisp2_RL_ef1α_ parasite values was set to 100%. Results show mean and standard deviations of three separate experiments (Unpaired student t-tests for each transgenic line to control were calculated, **p<0.01, ***p<0.001). (B) Luciferase activities of parasites transfected with various promoter truncations. Blood, oocyst, and 48h liver stages depicted. The FL/RL ratio of pFL_ef1α_RL_ef1α_ parasites at all stages was considered the baseline value and set to 100%. For better orientation, representative images of the different life cycle stages are shown (Wright stained blood smear, oocyst by transmitted light imaging, GFP-expressing HepG2 cell infected with mCherry-expressing *P*. *berghei* parasite).

Having determined liver stage-specificity of the *lisp2* promoter region *in vivo* and *in vitro*, we aimed to further characterize regulatory sequence elements in the promoter region. To extend our previous studies [11], various lengths of promoter regions were sub-cloned in the dual luciferase plasmid upstream of the firefly luciferase-encoding DNA sequence (Fig 4A) and transfected in *P*. *berghei* parasites similar to what has been described earlier [11]. After infection of HepG2 cells, the ratio of firefly to renilla luciferase expression was determined for all promoter region lengths at 48 hpi. The ratio of firefly/renilla (FL/RL) activity of the original promoter (-989/+1) in PbFL_lisp2_RL_ef1α_ parasites was set as 100% reference. Two constructs, (-775/+1 and -318/+1), have already been tested [11] and gave very similar results in the current study, confirming the robustness and reliability of this assay. Interestingly, truncating the promoter to -828/+1 did not significantly affect luciferase expression whereas any further deletion significantly reduced luciferase expression and thus indicates reduced promoter activity. It is important to note that the construct -594/+1 still allowed a strong expression whereas the regions -428/+1 and -318/+1 completely lost liver stage specific promoter activity, strongly suggesting an essential transcription enhancer element in the region between -594 and -428. Interestingly, in this region neither a typical enhancer binding site nor a typical ApiAP2 site have been identified (S3 Fig) strongly suggesting that other liver stage specific enhancer sites must exist.

Since the transcription start site (TSS) was localized at position -318 it was not surprising that with the -318/+1 construct no luciferase activity was detected, suggesting no promoter activity; also the constructs -989/-318 and -775/-318 were not active anymore confirming that the 5’ untranslated region (5’ UTR) is essential for gene expression.

Next we tested all different parasite strains for luciferase activity in different life cycle stages to see whether deletion of parts of the upstream region has an influence of stage specificity of the promoter (Fig 4B). Apart from the liver stage at 48 hpi, the blood stage and the mosquito stage (oocyst-infected midguts) were analyzed. For an easier comparison in all different life cycle stages we calculated the FL/RL ratio observed in PbFL_ef1α_RL_ef1α_, and set this value to 100%. With this approach we could also directly compare the liver stage specific promoter and the ef1α promoter, and we found that during the liver stage at 48 hpi the -989/+1 construct was about twice as strong as the already strong e1fα promoter. It is also remarkable that most constructs remained either liver stage specific or were silent in all stages. Only two constructs (-594/+1 and -428/+1) showed a clear and reproducible activity in oocysts. The fact that -428/+1 allowed some activity in oocysts but in none of the other stages suggests that it contains some oocyst-specific regulatory elements.

Together, it can be concluded that the most important regulatory element must be localized between -594 and -428 bp in terms of expression and of stage specificity. We hypothesize that additional regulatory elements might be localized in the more upstream regions -828 to -594, as indeed we identified various typical CAAT and TATA boxes in this region (S3 Fig). Nevertheless, it is important to note that to our knowledge, CAAT-binding regulatory elements have not been described so far for *Plasmodium parasites*, and their effect on specific promoters remains hypothetical.

### Substitution of the 5’UTR of the *lisp2* promoter region

Deletion of the entire 5’UTR is not the best method to define regulatory regions because the putative upstream-localized enhancer binding sites are shifted towards the start codon. To exclude the possibility that deletion analyses may have induced potential structural effects due to alteration of 5’UTR size and distance to the promoter, we decided to perform various substitution analyses to observe the effect of the 5’UTR in defining stage specificity. To determine whether the 5’UTR region is involved in regulation of expression, we searched for alternative 5’UTRs of similar length to the *lisp2* promoter 5’UTR (i.e. 318bp). By retaining the distance to the promoter, we intended to exclude potential structural effects influencing the promoter region. The 313bp 5’UTR of the alpha-tubulin 1 gene (PBANKA_041770), and the 322bp 5’UTR of the stage-specific antigen expressed apical membrane 1 (AMA1, PBANKA_091500), were selected by analyzing PlasmoDB ESTs and comparison with *P*. *falciparum* homolog mRNAs.

**Fig 5:**
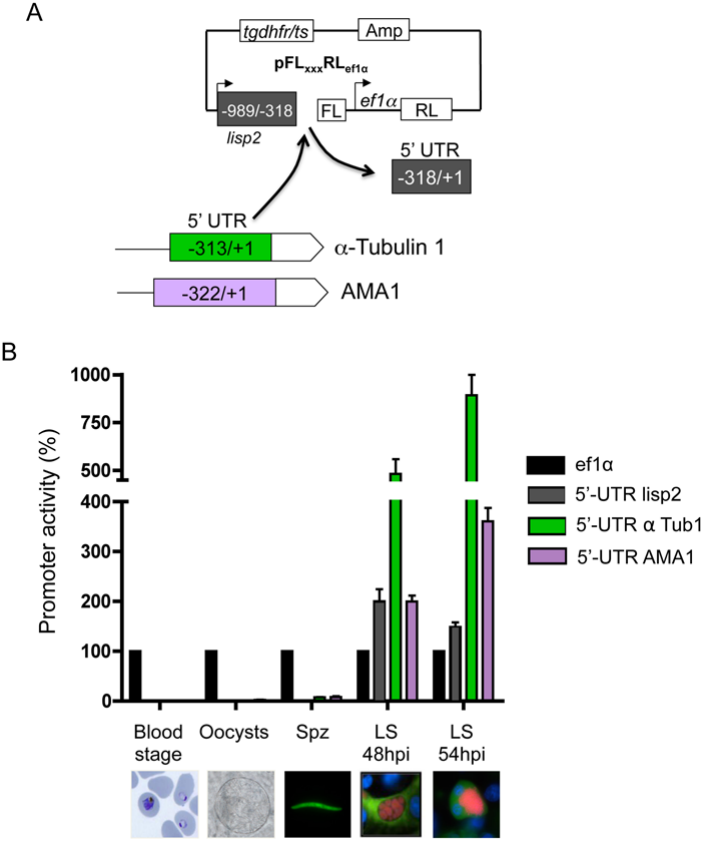
Analysis of the *lisp2* promoter activity upon 5’UTR substitutions in various life cycle stages. (A) Cloning strategy for the replacement of the 5’UTR of the *lisp2* promoter region. (B) Comparative activities of the *lisp2* promoter region, to those with α-Tubulin or AMA-1 exchanged 5’UTRs during all life cycle stages. HepG2 cells infected with *P*. *berghei* transgenic sporozoites and lysed at 48 and 54 hours post infection for luminescence measurements. The FL/RL ratio of PbFL_ef1α_RL_ef1α_ parasite lysates is set to 100%. Measurements were obtained in three independent experiments; means and SDs are shown.

The 5’UTR of the *lisp2* (-318/+1) promoter region was replaced by either the AMA1 or the alpha-tubulin 5’UTRs, fused to the remainder of the promoter, and cloned into the dual luciferase gene (Fig 5A). The resulting plasmids were transfected into *P*. *berghei* resulting in the PbFL_lisp2-αTub1_RL_ef1α_, and PbFL_lisp2-AMA1_RL_ef1α_ transgenic parasites. For consistency, and to establish a control for FL/RL determinations at all life stages, the ratio of FL/RL activity of PbFL_ef1α_RL_ef1α_ parasites expressing both renilla and firefly under the control of the constitutive promoter, were set to 100%. Substitution of the *lisp2* 5’UTR with the 5’UTR of the α-tubulin and AMA1 genes resulted in markedly altered firefly luciferase expression (Fig 5B). PbFL_lisp2-αTub_RL_ef1α_ parasites showed low firefly luciferase activity in the oocyst and sporozoite stages (2.5% and 8% respectively, compared to the constitutive promoter), as did PbFL_lisp2-AMA1_RL_ef1α_ parasites in the sporozoite stage (7.5%, compared to the constitutive promoter). Interestingly, both parasite strains did not show any firefly luminescence during the blood stage. However, during the liver stage development at 48 hpi, PbFL_lisp2-αTub_RL_ef1α_ showed a strong increase in luciferase activity (to 500% instead of 200% of the original promoter), which again increased almost by 2-fold towards merozoite formation (54 hpi) at the end of the hepatic phase. PbFL_lisp2-AMA1_RL_ef1α_ showed an activity comparable to the original promoter at 48 hpi but in contrast to the original promoter showed an increased activity at 54 hpi. The exchange of the 5’UTR could have transcriptional, as well as post-transcriptional effects, for instance through stabilizing effects on the mRNA, or increased translation rates. Further studies are needed to clarify this. Although such analyses are beyond the scope of this study, determination of the basis of the increased protein expression are particularly interesting as the observed effect was very impressive. Together, swapping the 5’UTR has a profound effect on luciferase expression but it remains to be determined whether this effect is at a transcriptional or rather at a post-transcriptional level.

Our data suggest that the liver stage-specific promoter region of *lisp2* can be used for various purposes. It offers the opportunity to drive expression of dominant-negative proteins to study protein function during the late liver stage and, perhaps even more importantly, to express parasite toxins to generate parasite vaccine strains attenuated during the late liver stage. In fact, recent studies have suggested that immunizations with genetically attenuated parasites that are attenuated in late liver stages, result in increased protection against challenge with fully virulent *Plasmodium* parasites and are thus more suitable for immunization [49]. Importantly, we previously generated a double-attenuated *Plasmodium* parasite, in which a bacterial pore-forming protein (perfringolysin) was expressed under the *lisp2* promoter, successfully leading to protection [50]. In conclusion, the *lisp2* promoter should greatly aid in our understanding of pre-erythrocytic *Plasmodium* biology as well as allowing attenuation of parasites at a very defined time point of liver stage development, thus facilitating the generation of live attenuated vaccine strains. Furthermore, the promoter holds the potential of being used as part of an inducible system to study the *Plasmodium* liver stage.

## Supporting Information

S1 Fig Cell line measurement differences for *in vitro* imaging. PbFL_lisp2_RL_ef1a_ sporozoites were used to infect HepG2, Hepa1-6 and Huh7 cells. 40 hours post-infection cells were lysed by passive Lysis 5x buffer and mechanical disruption, and luminescence measured using the Dual Luciferase reporter assay system. The (A) renilla and (B) firefly luminescence expressed as RLUs, of each cell line is shown. Although the ratio between substrates is maintained, the absolute luminescence values for each substrate differ significantly between the different cell lines.
(TIFF)

S2 Fig Relative parasitemia and renilla bioluminescence values during blood-stage infection. Mice were infected with PbFL_lisp2_RL_ef1a_ parasites but only ef1α promoter activity as shown by renilla luminescence was assessed during blood stage development. It coincides with increased parasite burden (measured by Wright’s stain) over the course of 170h (7 days) following intravenous injection of sporozoites.
(TIFF)

S3 Fig Identified enhancer binding elements in the *lisp2* promoter region. The transcription start site (TSS) was identified 318 bp upstream of the start codon. Putative CAAT, TATAA as well as TATAA like boxes within the promoter region are indicated. In addition, the sporozoite-specific enhancer binding element CATGCCAN [51] and two ApiAP2 binding elements have been identified. The motifs shown as D1 and D2, are putative sites predicted based on *in silico* analyses using the motifs identified for the *P*. *falciparum* ortholog [11]. Evaluated deletions are also included in the diagram as arrows and significant decrease in promoter activity by the deletion are indicated (**P<0.01; *** P<0.001).
(TIFF)

S1 Table Optimization of the dual bioluminescence system for *in vivo* imaging of *Plasmodium* liver stages. The dual luciferase bioluminescence system for *P*. *berghei* imaging in the liver required optimal pairing of each specific luciferase and a set of substrates; identification of factors involved in signal quenching or enhancement; identification of the optimal route of administration; animal physiological factors that must remain constant for optimal imaging; and potential extrinsic factors influencing imaging and ultimately signal detection.
(DOCX)
