# Supplementary figures and images for: In Vivo and In Vitro Characterization of a Plasmodium Liver Stage-Specific Promoter
Source: PLoS One. 2015 Apr 15;10(4):e0123473. doi: 10.1371/journal.pone.0123473 (PMC4398466; doi:10.1371/journal.pone.0123473)

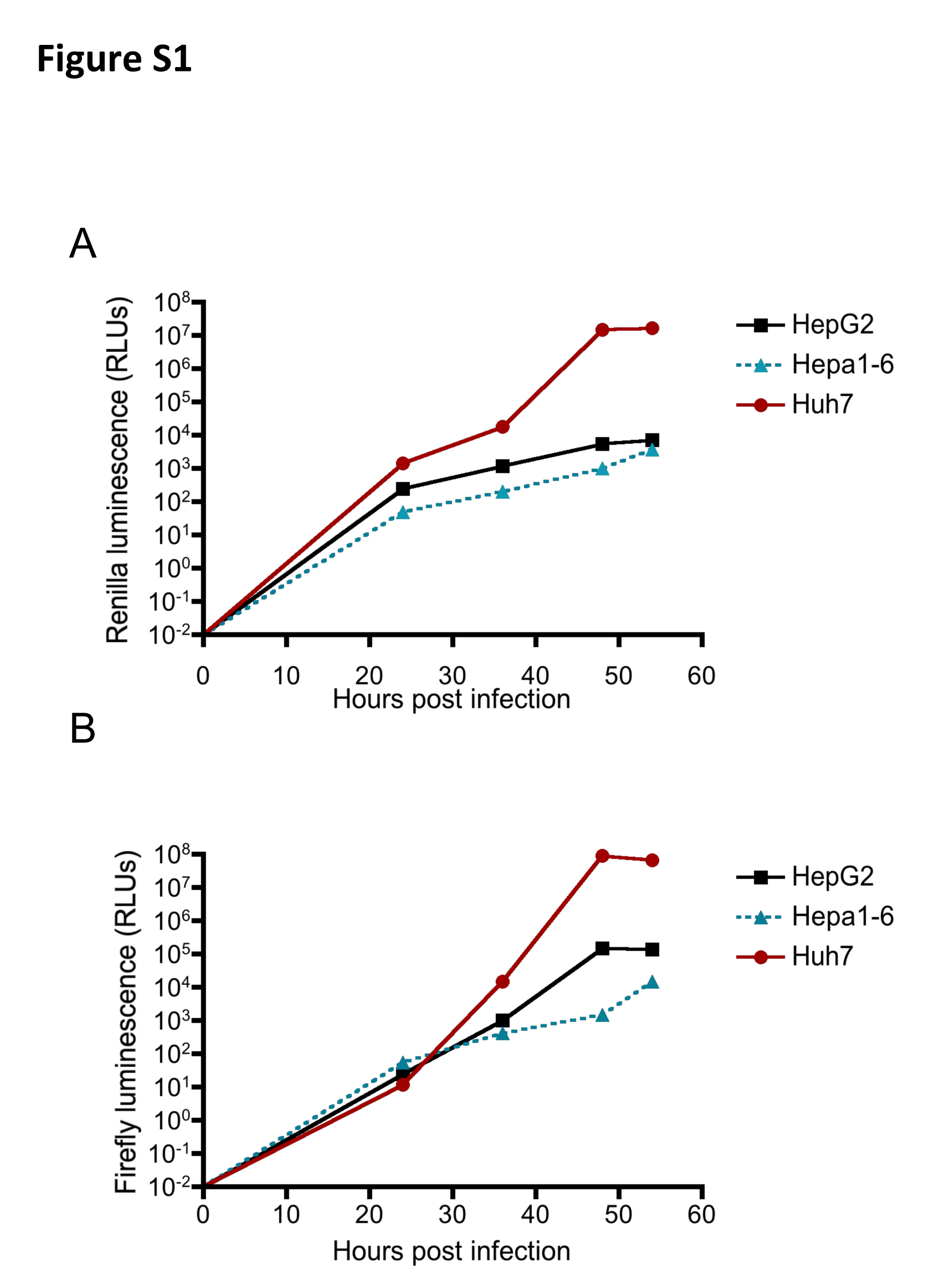

Supplement: S1 Fig — PbFLlisp2RLef1a sporozoites were used to infect HepG2, Hepa1-6 and Huh7 cells. 40 hours post-infection cells were lysed by passive Lysis 5x buffer and mechanical disruption, and luminescence measured using the Dual Luciferase reporter assay system. The (A) renilla and (B) firefly luminescence expressed as RLUs, of each cell line is shown. Although the ratio between substrates is maintained, the absolute luminescence values for each substrate differ significantly between the different cell lines. (TIFF) [file pone.0123473.s001.tiff]

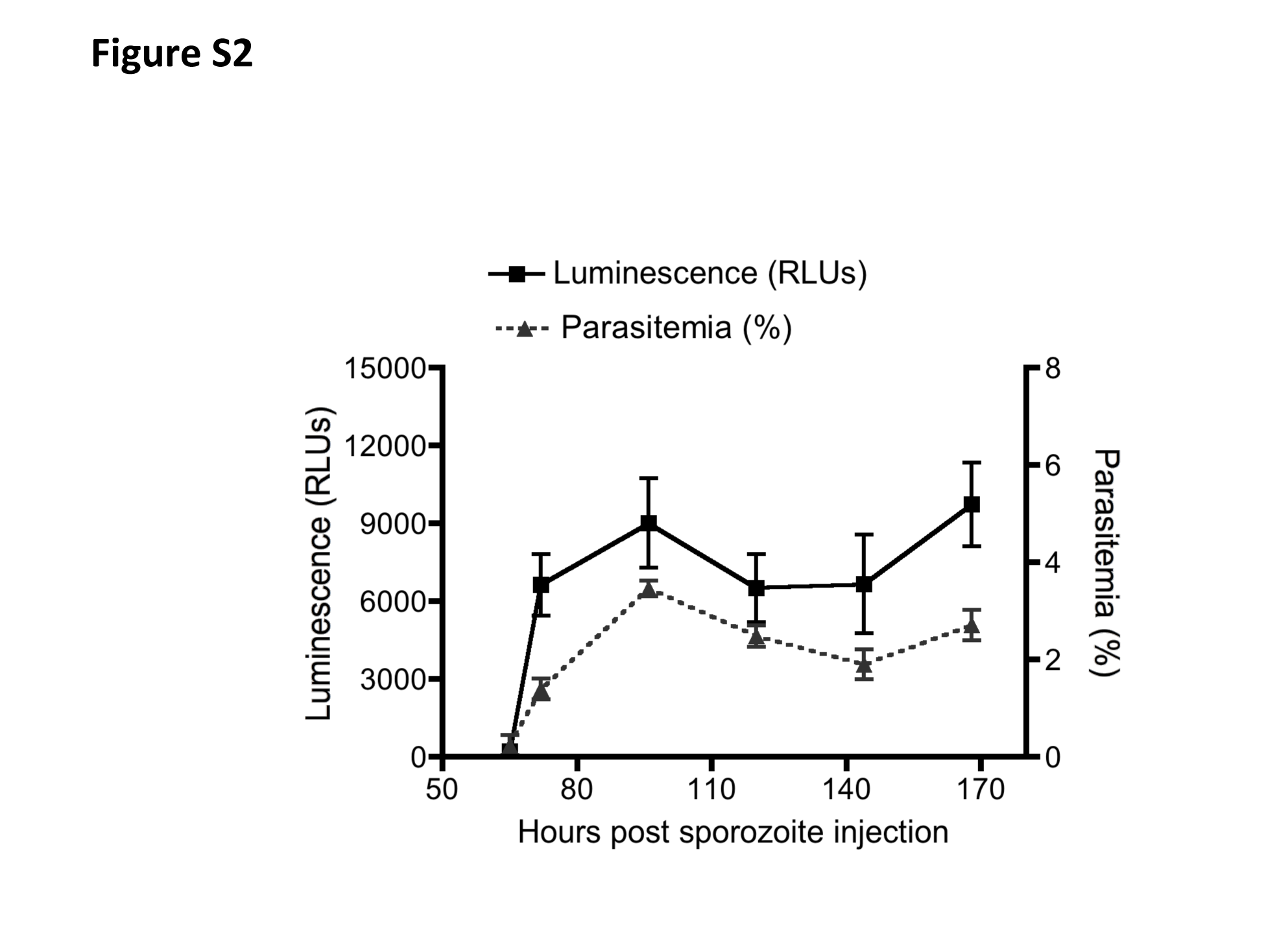

Supplement: S2 Fig — Mice were infected with PbFLlisp2RLef1a parasites but only ef1α promoter activity as shown by renilla luminescence was assessed during blood stage development. It coincides with increased parasite burden (measured by Wright’s stain) over the course of 170h (7 days) following intravenous injection of sporozoites. (TIFF) [file pone.0123473.s002.tiff]

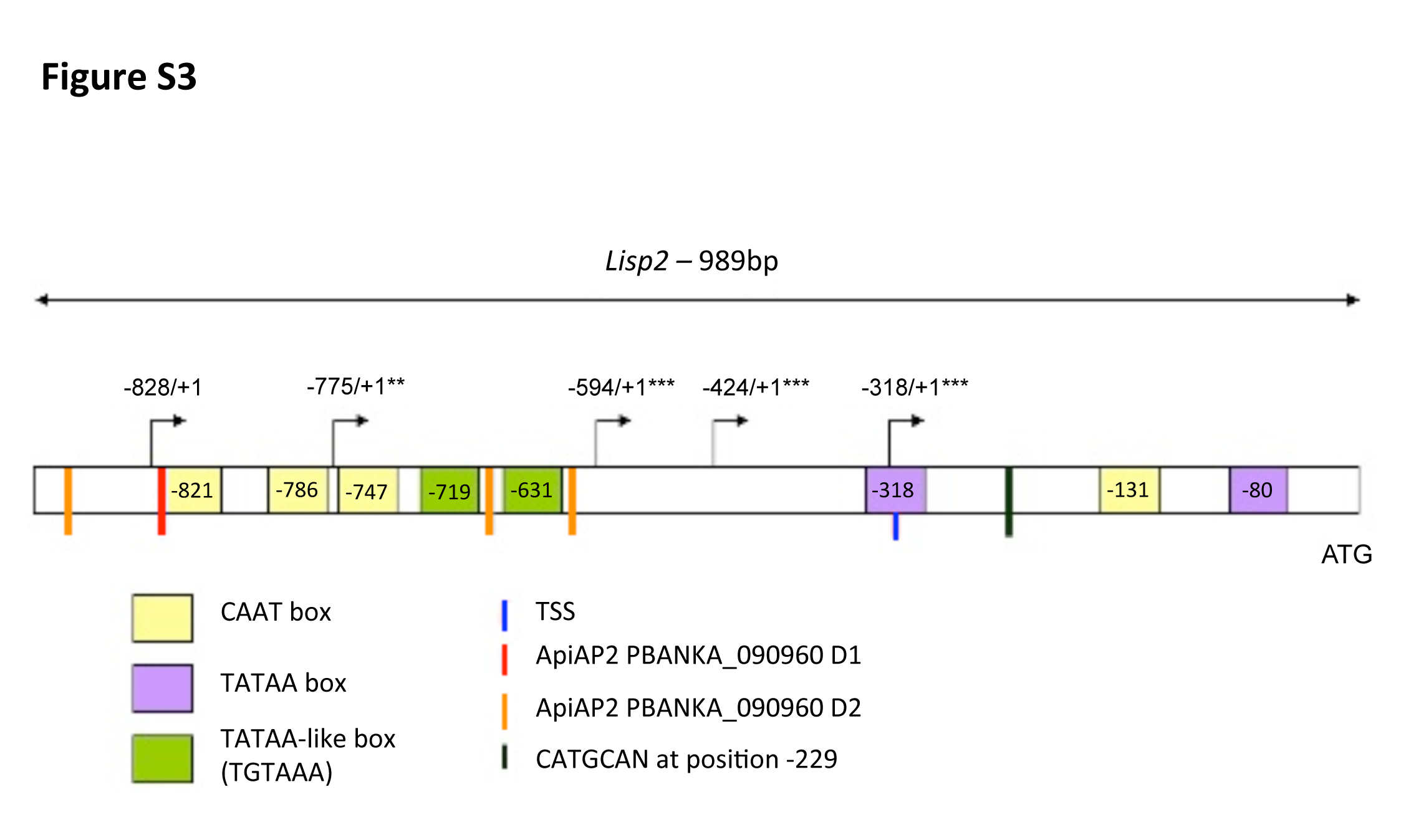

Supplement: S3 Fig — The transcription start site (TSS) was identified 318 bp upstream of the start codon. Putative CAAT, TATAA as well as TATAA like boxes within the promoter region are indicated. In addition, the sporozoite-specific enhancer binding element CATGCCAN [51] and two ApiAP2 binding elements have been identified. The motifs shown as D1 and D2, are putative sites predicted based on in silico analyses using the motifs identified for the P. falciparum ortholog [11]. Evaluated deletions are also included in the diagram as arrows and significant decrease in promoter activity by the deletion are indicated (**P<0.01; *** P<0.001). (TIFF) [file pone.0123473.s003.tiff]
