# Supplementary material for: In Vivo and In Vitro Characterization of a Plasmodium Liver Stage-Specific Promoter
Source: PLoS One. 2015 Apr 15;10(4):e0123473. doi: 10.1371/journal.pone.0123473 (PMC4398466; doi:10.1371/journal.pone.0123473)
Supplement: S1 Table — The dual luciferase bioluminescence system for P. berghei imaging in the liver required optimal pairing of each specific luciferase and a set of substrates; identification of factors involved in signal quenching or enhancement; identification of the optimal route of administration; animal physiological factors that must remain constant for optimal imaging; and potential extrinsic factors influencing imaging and ultimately signal detection. (DOCX) [file pone.0123473.s004.docx]

**Table S1**

| **Bioluminescence feature** | **Drawbacks/limitations** | **Optimization steps** |
| --- | --- | --- |
| Enzyme-substrate pairing: Renilla | Coelenterazine (CZ), the substrate for renilla luciferase, has low availability and peak emission at lower wavelengths (480nm). Native coelenterazine leads to high signal-to-noise ratio in i.p. or unsuccessful i.v. injections. The kinetics and half life of the enzyme are extremely fast. May lead to unstable signal. | Various CZ analogs were used and kinetics characterized for liver imaging. ViviRen had a 120-fold better signal than native CZ, with lower background noise. Coelenterazine-h (in RediJect format) showed 50-fold higher signal and low signal-to-noise ratio. Due to the high cost of ViviRen for a large set of experiments, Coelenterazine-h available as RediJect was the method of choice. |
| Enzyme-substrate pairing: Firefly | D-luciferin, the substrate for firefly luciferase, produces light in the 550-570nm range, with a very high quantum yield. Its uptake is ATP and oxygen-dependent, limiting its use in hypoxic environments or tissues with high cell death rates. Various substrates exist, based on sodium and potassium salts. Reconstitution variations may lead to significant differences among luminescence measurements. | We found no difference in luminescence upon use of sodium or potassium-based D-luciferin. To diminish potential variability with salt reconstitution, the RediJect D-luciferin format was chosen. |
| Signal quantification | Measurements using the dual luciferase system may be subject to substrate/luciferase-dependent variations in vivo. | A control comparing Renilla and firefly luciferases, both under the constitutive ef1a promoter, was included. |
| Administration route | Variations in organ distribution and uptake have been reported for firefly luciferase, when different administration routes, namely subcutaneous, i.p., or i.v. routes are used. I.p. injections are believed to result in prolonged organ uptake of D-luciferin.  Renilla luciferase possesses flash-type kinetics, which requires obligatory i.v. injection of the substrate. | Experiment replicates were carried out with different administration routes in the same animal until the signal of the initial injection quenched. D-luciferin was administered i.p., given the better availability we observed in the liver.  To perform renilla measurements during the liver stage, optimization was very critical. Finally, the signal was measured within 20s of i.v. injection. Failed i.v., i.p., and s.c. injections resulted in very high signal-to-noise ratios especially in the abdominal area. |
| Exogenous factors: luciferase modulators | Small molecule inhibitors of firefly luciferase have been characterized. Different ones have been described for renilla luciferase. Among the group of modulators affecting firefly luciferase are anaesthetics, including isofluorane. While anaesthetics have an effect on firefly luciferase, a similar effect for renilla has not been found. | Measurements were performed in animals anaesthetized with the following anaesthetic combinations: xylazine + ketasol; midazolam + ketasol. Anaesthesia with all components showed similar results.  To standardize measurements, exposure times and rates to isofluorane were determined.  Next generation in vivo luminescence systems are expected to enable measurements in real time, overcoming the need for anaesthesia. Controls in our study included mice infected with firefly and renilla under the constitutive ef1a promoter. |
| Animal physiology (external factors) | pH, temperature and a range of other physiological factors may affect the bioluminescent signal. | All mice in any determined group, were kept under the same conditions before, and throughout the experiment. During anaesthesia, the temperature of the surfaces in the induction chamber, was kept at 35°C. |
| Promoter activity | Extrinsic factors have been reported to impact different reporters, as is the modification of promoter regions to drive the expression of luciferases. | Promoter activities in native conditions were compared by qRT-PCR of *in vitro* hepatocyte cultures, and in organ extracts. |
